# Cadmium adsorption performance and mechanism from aqueous solution using red mud modified with amorphous MnO_2_

**DOI:** 10.1038/s41598-022-08451-2

**Published:** 2022-03-15

**Authors:** Yin Pang, Cong Zhao, Yao Li, Qin Li, Xiang Bayongzhong, Daoping Peng, Tao Huang

**Affiliations:** Faulty of Geosciences and Environmental Engineering, Southwest Jiaotong University, Chengdu, 610031 China

**Keywords:** Environmental sciences, Materials science

## Abstract

In this study, red mud modified by manganese dioxide(MRM) was utilized as an adsorbent to effectively remove Cd^2+^ from aqueous solution. The characteristics were analysed by SEM–EDS, XRD, BET, FTIR and XPS. Different factors that affected the Cd^2+^ removal on MRM, such as dosage, initial pH, initial Cd^2+^ concentration, were investigated using batch adsorption experiments. Simultaneously, the adsorption kinetics, adsorption isotherms and adsorption thermodynamics of Cd^2+^ were also investigated using adsorption experiments data. The characterization results showed that MRM had a rougher, larger specific surface area and pore volume (38.91 m^2^ g^−1^, 0.02 cm^3^ g^−1^) than RM (10.22 m^2^ g^−1^, 0.73 cm^3^ g^−1^). The adsorption experiments found that the equilibrium adsorption capacity of MRM for Cd^2+^ was significantly increased to 46.36 mg g^−1^, which was almost three times that of RM. According to the fitting results, the pseudo-second-order kinetic model described the adsorption process better than the pseudo-first-order kinetic model. The Langmuir model fitted the adsorption isotherms well, indicating that the adsorption process was unimolecular layer adsorption and the maximum capacity was 103.59 mg g^−1^. The thermodynamic parameters indicated that the adsorption process was heat-trapping and spontaneous. Finally, combined XPS and FTIR studies, it was speculated that the adsorption mechanisms should be electrostatic attachment, specific adsorption (i.e., Cd–O or hydroxyl binding) and ion exchange. Therefore, manganese dioxide modified red mud can be an effective and economical alternative to the removal of Cd^2+^ in the wastewater treatment process.

## Introduction

Heavy metal ions are extremely toxic and carcinogenic, seriously endangering the safety of water in today's world^1^. Cadmium (Cd) is one of the most toxic elements for humans, which is widely used in various industrial activities, inculding alloy manufacturing, smelting, electroplating, pigments, plastics and battery manufacturing^2^. Cd^2+^ could enter the human body through the food chain and deposit in various tissues of the body, which would lead to serious diseases and endanger human health and life^3^. According to the joint regulations of the World Health Organization (WHO), the U.S. Environmental Protection Agency (EPA) and the European Public Health Alliance (EPHA), the maximum amount of Cd^2+^ contamination in drinking water should not exceed 0.003 mg L^−1^^4^. Currently, the main technologies for Cd^2+^ removal include chemical precipitation, membrane filtration, adsorption, ion exchange, electrodialysis, and biological methods. Among these treatment methods, adsorption has proved to be an effective and economical method for the removal of Cd^2+^ from the aqueous environment because of its simplicity, ease of operation, low cost, and excellent adsorption properties^5^. The adsorption method is mainly used to remove heavy metals by adsorption through the physicochemical action of heavy metal ions by the internal porous structure and surface active sites of the adsorbent material. Some adsorbents can be regenerated and recycled through a suitable desorption process. After years of research, researchers have developed a variety of adsorbent materials with strong adsorption capacity for heavy metals^6,7^. However, the practical application of most adsorbent materials is very limited due to the cost and time consuming limitations. Therefore, the preparation of low-cost and efficient heavy metal adsorbents remains a research priority in the field of wastewater treatment.

Over the past few years, red mud (RM), a solid waste generated during the production of the alumina industry, has received several attentions due to its huge pile and resource utilization difficulties^8–11^. Red mud is strongly alkaline, has large porosity and high dispersibility, which make it have a good adsorption effect on metal cations^12^. Simultaneously, its application as an adsorbent in the field of heavy metal wastewater treatment can realise both environmental and economic benefits^13^. However, the final removal effect could not reach the specified concentration limit due to the low adsorption capacity and strength structure of red mud. To further enhance the adsorption performance of red mud, scholars have adopted different activation methods, such as acidification^14,15^, heating^16^, soaking in sea water^17^. For example, some researchers have adopted simple acid activation and then precipitated red mud with ammonia to explore the possibility and adsorption rate of Cd^2+^ removal in water^18^. In addition, it is found that the adsorption stability of red mud for Cd^2+^ treated at 500 °C is almost twice that of the original red mud via studying the effect of heat treatment temperature on the adsorption capacity of Red mud for Cd^2+^^16^. However, the aforementioned modification methods are either costly to modify or have other problems, such as consuming too much thermal energy, difficult to perform, or low adsorption efficiency. One of the solutions to these problems is the use of metal compounds to modify the structure and surface area of the red mud, thus enhancing the adsorption properties.

Manganese dioxide (MnO_2_), an environmentally friendly metal oxide, has the advantages of large specific surface area, high surface activity, low price and easy availability^19^. More importantly, MnO_2_ has stable chemical properties and specific redox effects. MnO_2_ is an excellent adsorbent because of its ability to form complexes with heavy metal ions (e.g., Cd^2+^, Cu^2+^, Zn^2+^, and Pb^2+^) and good chemical stability under basic and acidic conditions^20,21^. For instance, amorphous hydrated manganese dioxide (HMO) had the selective removal of three metal ions (Pb^2+^, Cd^2+^, and Zn^2+^) and it was concluded that the adsorption of the three metal ions could be related to the inner sphere complexation^20^. Hydrated manganese dioxide had high efficient Cd^2+^ adsorption performance, and the maximum adsorption capacity for Cd^2+^ at 25 °C was 168.36 mg g^−1^, which was significantly higher than most heavy metal adsorption materials^22^. Unfortunately, the poor mechanical stiffness, excessive pressure drop and particular agglomeration limit the practical application of MnO_2_. MnO_2_ can be loaded on carriers with a large surface area to enhance adsorption and dispersibility^23,24^. Most materials with large surface areas are difficult and costly to fabricate, making them hard to scale up and apply. It is crucial to find low-cost carrier materials for MnO_2_, and bulk industrial solid waste, red mud, demonstrates its advantages in this regard.

The purpose of this study is to explore the behavior and mechanism of using amorphous MnO_2_ modified red mud to enhance the removal of cadmium. The manganese dioxide modified red mud (MRM) was prepared by the redox reaction between potassium permanganate and manganese sulfate for Cd^2+^ adsorption. The load level MnO_2_ could be controlled by changing the amount of red mud during the experiment. The introduced MnO_2_ is an amorphous phase, and the amorphous oxides have better adsorption properties than their crystals^25^. Then, it was investigated the effects of different factors on the adsorption of Cd^2+^. Besides, the adsorption kinetics and isotherms are discussed to explore the process of Cd^2+^ adsorption in detail and finally investigated the adsorption mechanism by Fourier transform infrared spectroscopy (FTIR) and X-ray photoelectron spectroscopy (XPS).

## Results and discussion

### Characterizations of adsorbents

**Figure 1:**
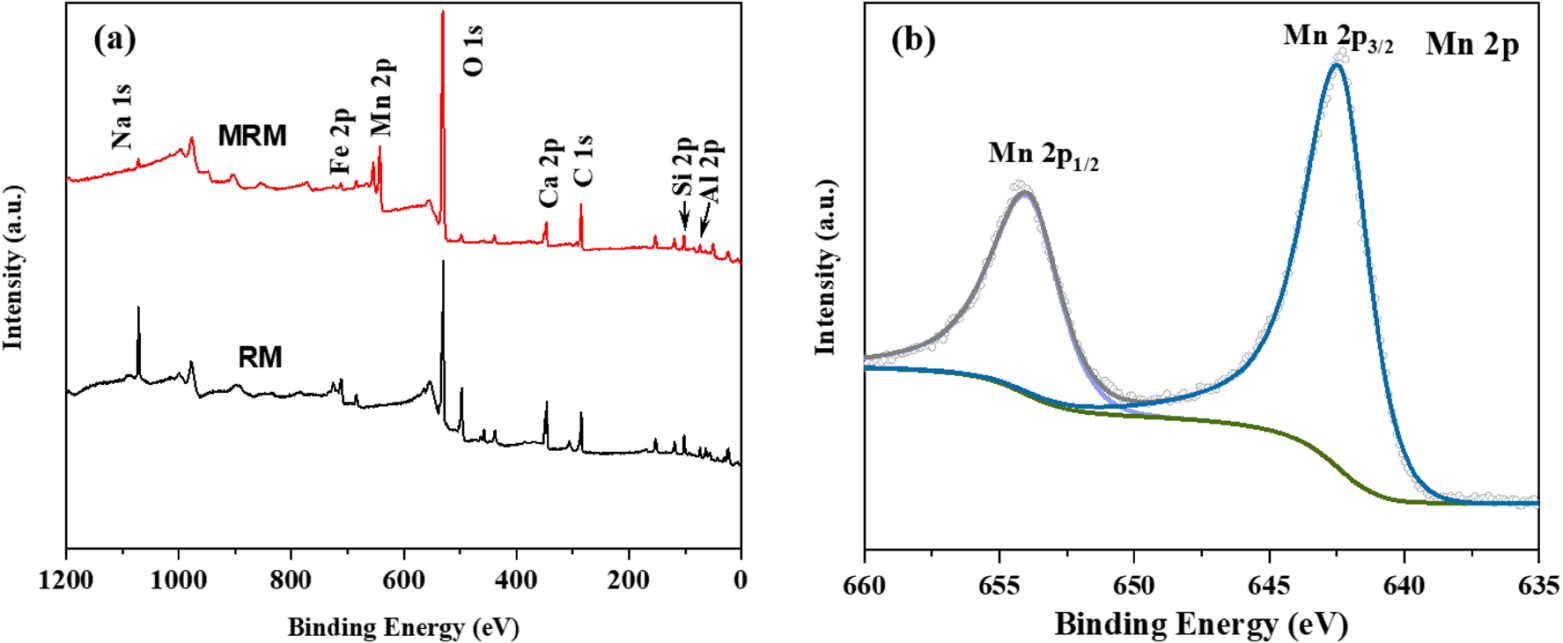
Full-scan XPS spectra of (**a**) RM and MRM and (**b**) Mn 2p spectra of MRM.

XPS was used to characterise the RM and MRM surface elements. An increase in the intensity of the Mn 2p peak at 643.08 eV was observed in Fig. 1a, and the acceptable spectrum of Mn 2p is shown in Fig. 1b at 642.42 eV and 653.95 eV for Mn 2p_3/2_ and Mn 2p_1/2_, respectively. The binding energy difference between Mn 2p_1/2_ and Mn 2p_3/2_ is 11.54 eV, indicating that the Mn in MRM valence state is mainly Mn^4+^, which also indicates that the main form of manganese oxide on the load is MnO_2_^26,27^.

**Figure 2:**
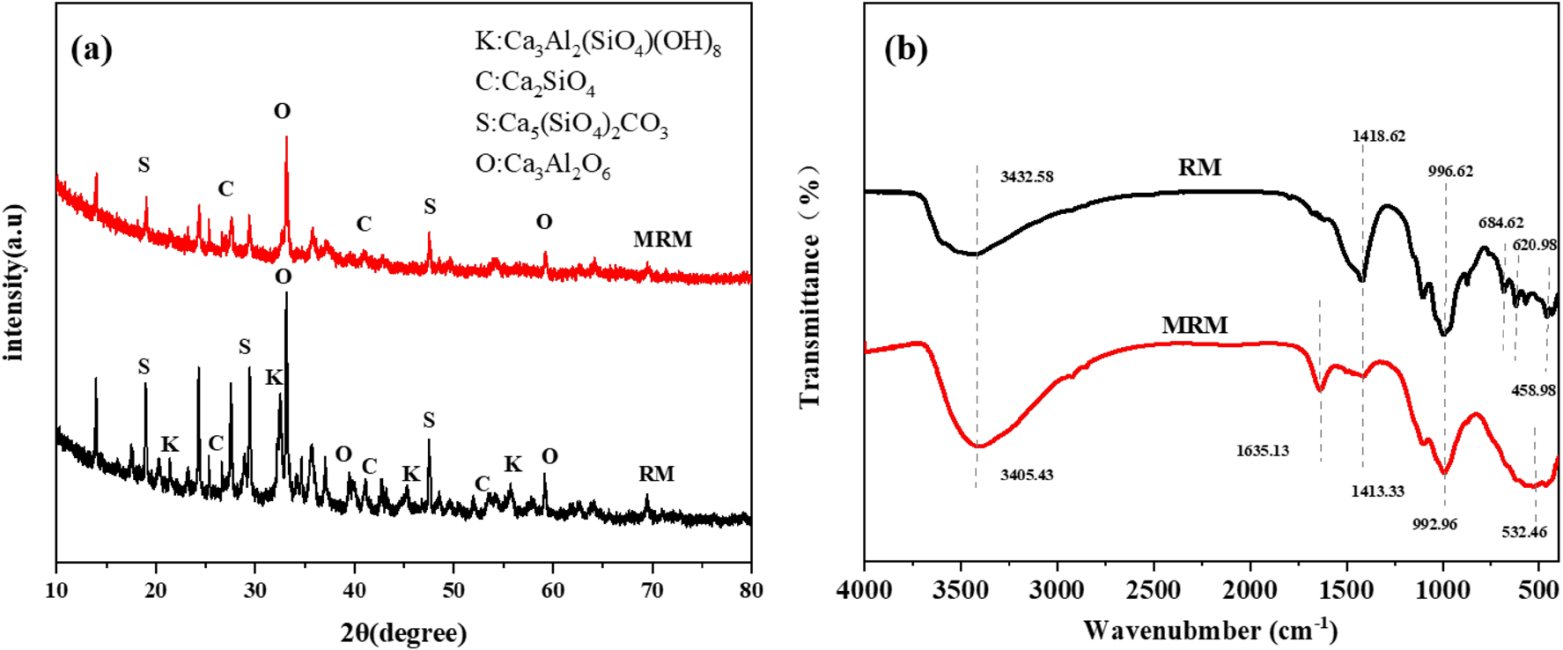
The XRD (**a**) and FTIR (**b**) of RM and MRM.

The mineral composition of RM before and after loading was analysed by XRD, and the results are shown in Fig. 2a. The main phases of RM are minerals dominated by Si, Ca, and Al, such as Ca_3_Al_2_(SiO_4_)(OH)_8_(Katoite, Si-rich), Ca_2_SiO_4_(Calcium Silicate), Ca_5_(SiO_4_)_2_CO_3_(Spurrite) and Ca_3_Al_2_O_6_(Calcium Aluminum Oxide). After the loading of MnO_2_, the peak intensities of the characteristic peaks contained in RM all weakened and some even disappeared, but no new characteristic peaks were generated. This may be because the MnO_2_ loading of the amorphous phase weakened the intensity of the diffraction peaks of the other crystals^28^. It can be inferred that the MnO_2_ loaded on the surface of the red mud in this study is a non-chemo-metric amorphous phase, which is possibly due to the change in the crystalline structure of the manganese dioxide during the preparation of the material^29^.The FTIR spectra of RM and MRM at wavelengths of 400–4000 cm^−1^ were analysed and the results are shown in Fig. 2b. It can be seen from the figure that the diffraction peaks of each functional group changed significantly after the loading of MnO_2_ and new characteristic peaks appeared. A solid and broad absorption band appeared at 3432.58 and 3405.43 cm^−1^ for RM and MRM, respectively, which corresponds to the O–H stretching vibration of the hydroxyl group on the surface of the samples^30,31^. The band of MRM at 1635.13 cm^−1^ is attributed to the O–H stretching vibrations in Mn-OH^20,32^. The band at 1418.62 cm^−1^ appearing in RM is attributed to CO_3_^2+^,proving that RM contains many carbonate compounds^33,34^. However, the CO_3_^2+^peak in the MRM shifts and decreases in intensity after loading MnO_2_. The peaks appearing at 996.62 and 992.96 cm^−1^ are caused by O–Si–O stretching vibrations^35,36^. The presence of Al–O stretching vibrations (684.62 cm^−1^), Si–O–Al stretching vibrations (620.98 cm^−1^) and Fe–O stretching vibration (458.98 cm^−1^) existing in RM disappeared in the MRM^37^, while a clear absorption peak at 532.56 cm^−1^ appeared in the MRM. This band at 532.56 cm^−1^ indicates the presence of Mn–O stretching vibratio^26^, further verifying the successful loading of MnO_2_. In conclusion, MRM has rich surface functional groups, which makes MRM have vigorous chemisorption activity.

**Figure 3:**
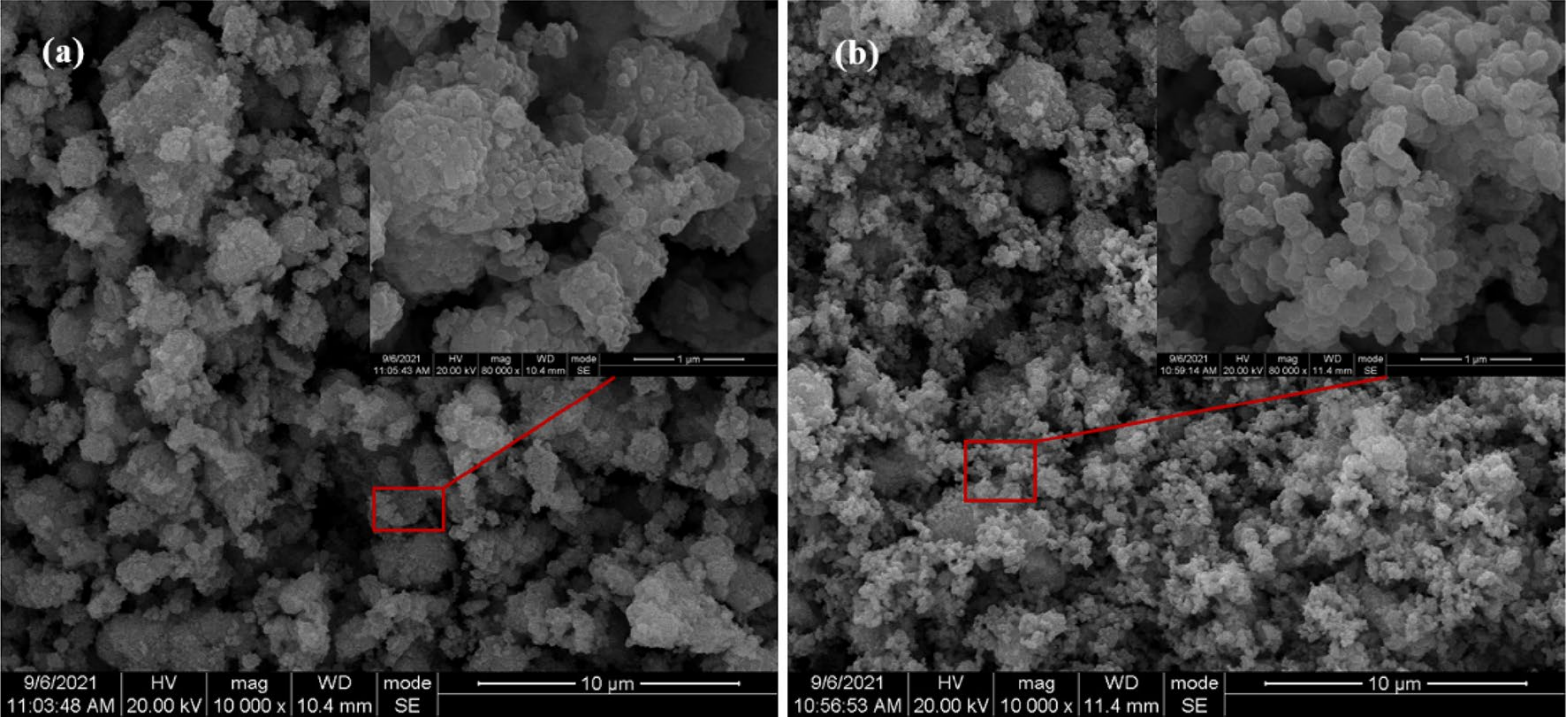
The SEM micrographs of RM (**a**) and MRM (**b**) at different magnifications.

**Figure 4:**
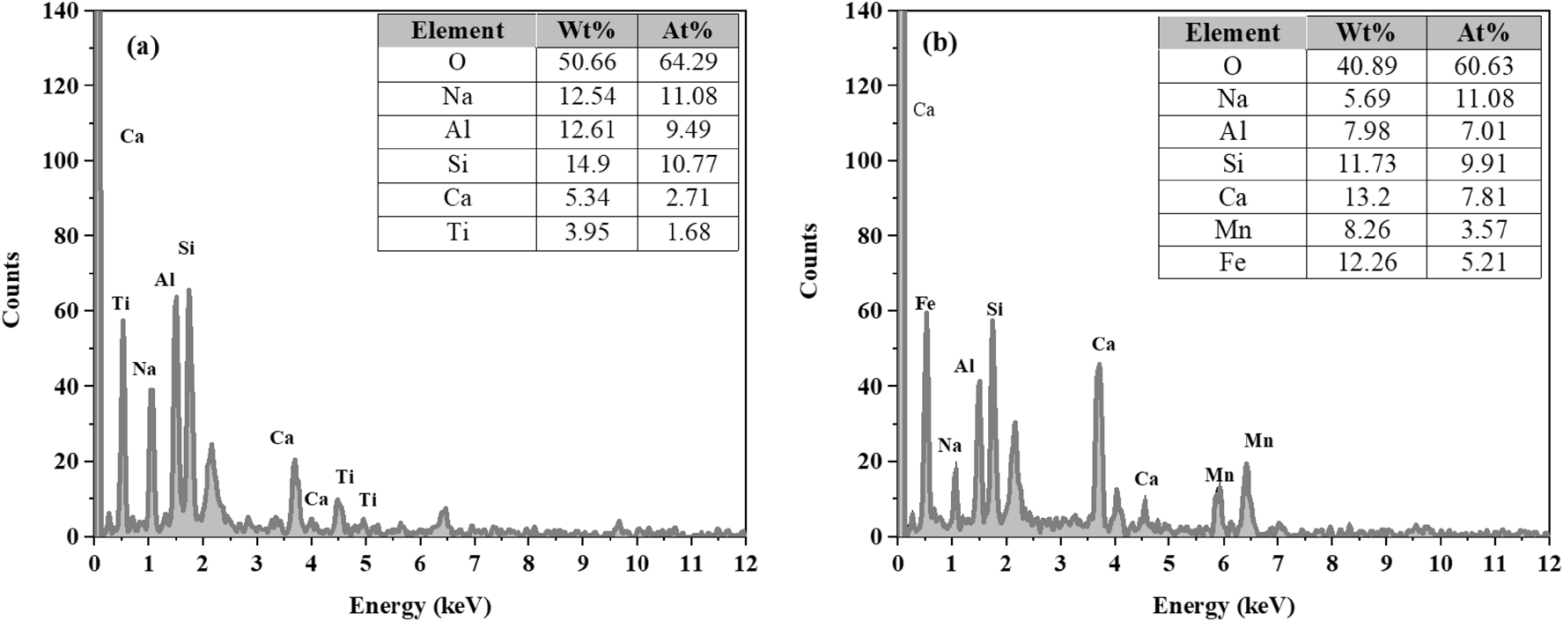
EDS analysis of (**a**) RM and (**b**) MRM.

The SEM micrographs of RM and MRM are shown in Fig. 3. It can be seen that the RM (Fig. 3a) surface shows the irregular distribution and the presence of large particle agglomerates and lamellar particles with many irregular small particles in their gaps. After MnO_2_ loading (Fig. 3b), MRM was obviously covered by MnO_2_. Its surface was covered by many spherical, and flocculent particles appeared, showing a three-dimensional porous structure, which in turn provided more adsorption sites on the surface, pore channels and interlayer domains, thus improving the adsorption performance of MRM. Comparing the EDS energy spectra before and after modification (Fig. 4a,b), the content of manganese in the original red mud was not detected, but the content of main metal elements in the red mud changed after MnO_2_ loading. The increase of Mn content also indicated that the red mud had been successfully loaded with MnO_2_.

**Figure 5:**
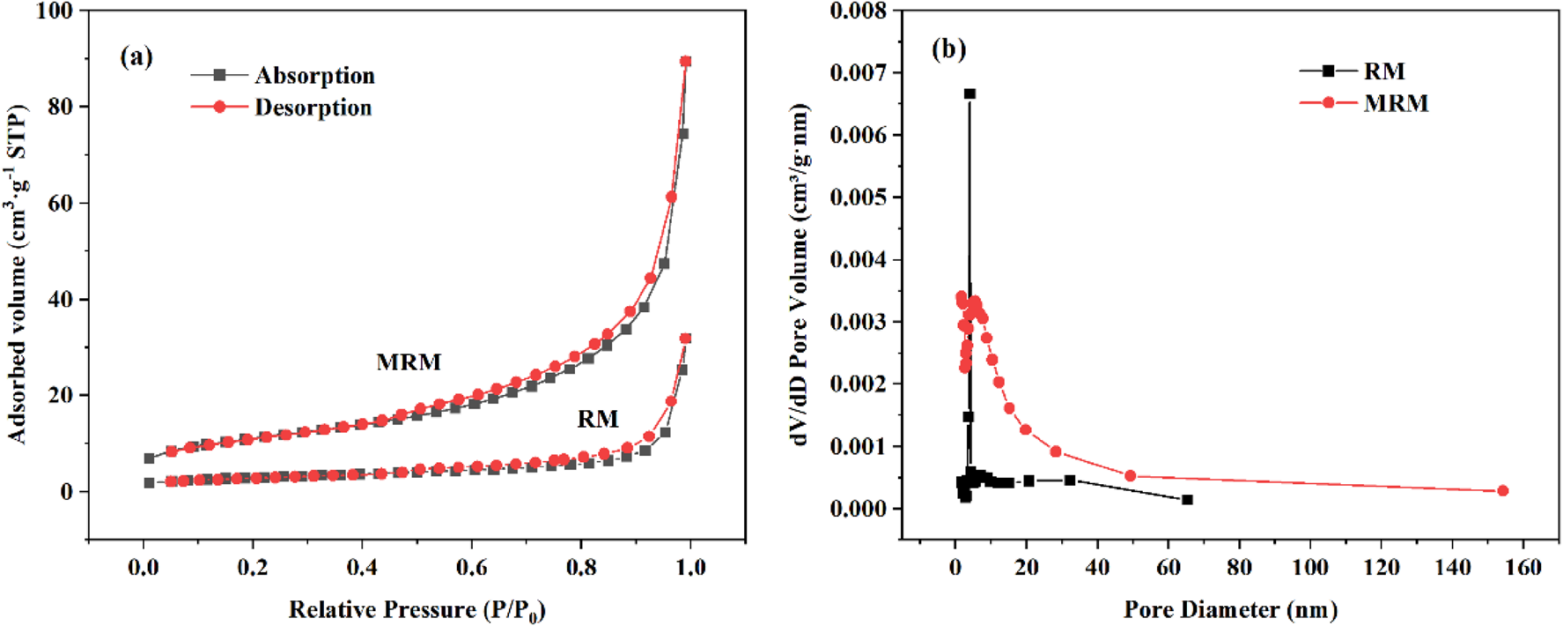
The N_2_ adsorption–desorption curves (**a**) and pore size distribution (**b**) of the RM and MRM.

Porosity and pore size distribution of RM and MRM were determined by nitrogen adsorption–desorption experiments, and the results are shown in Fig. 5. According to the standard of International Union of Pure and Applied Chemistry (IUPAC), the adsorption–desorption isothermal curves of both RM and MRM are of type VI (Fig. 5a). Both showed obvious hysteresis back loops due to capillary condensation, indicating the existence of mesoporous structures inside. Meanwhile, the closed hysteresis loops are similar to the H3-type hysteresis loops, indicating that the pore structure is very irregular. The specific surface area and pore size were calculated based on the BET model and the BJH model, respectively, and the results are shown in Table 1. The apparent increase in the specific surface area of MRM is due to the formation of manganese oxides on its surface, which is consistent with the successful loading of MnO_2_ as shown in the SEM and EDS energy spectra. However, the average pore size of MRM is smaller than that of RM, which is since the loading of MnO_2_ changes RM from dispersed particles to more dense particles and the number of pores increases. The pore size distribution curves (Fig. 5b) also show that the pore size distribution of MRM is broader and the number of pores increases. In addition, the increase in pore capacity and the number of pores facilitates the diffusion of metal ions into the interior of the adsorbent, which is also one of the advantages of MRM.

**Table 1:** BET surface area, pore volume and average pore diameter of the RM and MRM.

Samples	Surface area (m^2^ g^−1^)	Pore volume (cm^3^ g^−1^)	Average pore diameter (nm)
RM	10.2212	0.018292	22.1247
MRM	38.9082	0.72686	15.6217

### Adsorption study

#### Effect of different ratios of manganese dioxide to red mud

**Figure 6:**
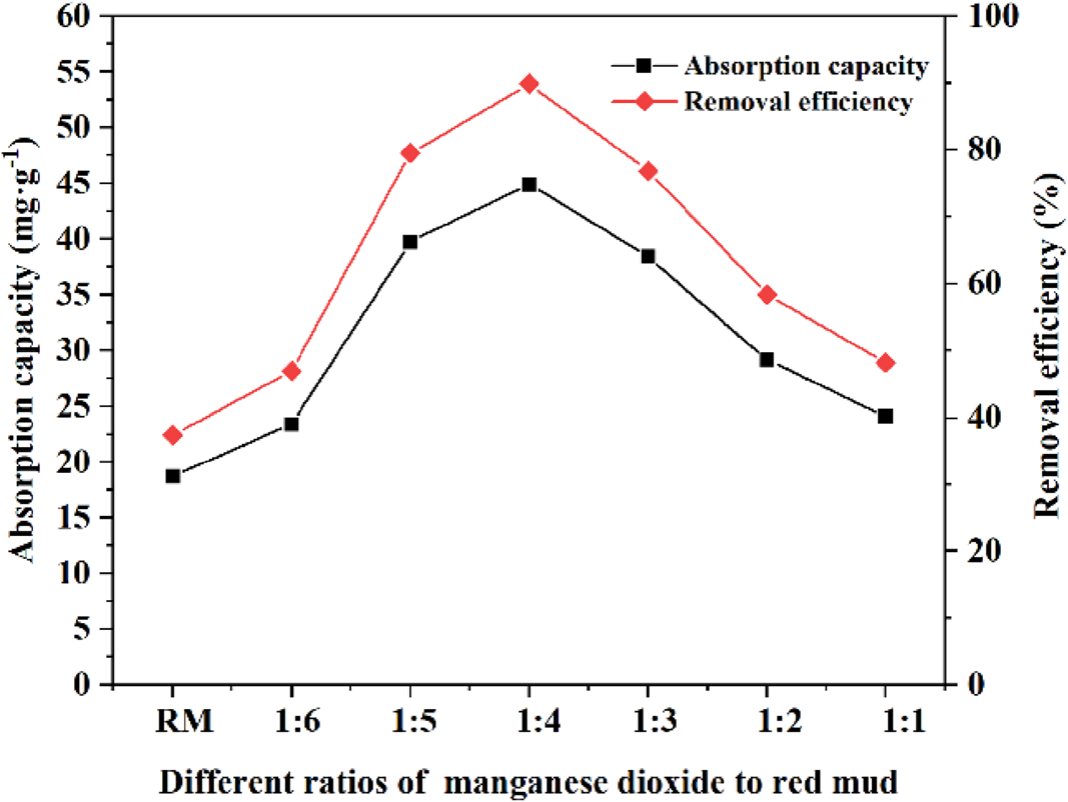
Effect of different ratios of manganese dioxide to red mud on adsorption of MBR at pH = 6, T = 25 °C, dosage = 1 g L^−1^, *t* = 240 min and initial Cd^2+^ concentration = 50 mg L^−1^.

From Fig. 6, it was found that the different ratios of MnO_2_ and red mud had essential effects on the adsorption performance of MRM. The equilibrium adsorption capacity of the original red mud for Cd^2+^ was 18.73 mg g^−1^; when the ratio of manganese dioxide to red mud was 1:4, the equilibrium adsorption capacity of the generated MRM for Cd^2+^ in solution reached the highest, 44.93 mg g^−1^, which was 26.20 mg g^−1^ higher than that of the original red mud for Cd^2+^; the equilibrium adsorption capacities of the other five ratios were also higher than that of the original red mud. The improved adsorption capacity of the modified red mud was attributed to the surface loading of MnO_2_. The loading of MnO_2_ on the red mud resulted in a higher surface activity, giving it a high adsorption affinity for the metal cation Cd^2+^. In addition, because the red mud material is a good carrier, MnO_2_ can be better loaded and dispersed on its surface, enhancing the composite's adsorption performance^38^. When the ratio of MnO_2_ to red mud was 1:1, the adsorption effect was not as good as the modified red mud with a ratio of 1:4. This may be due to the deposition of excess MnO_2_ on the surface of the red mud, which leads to pore blockage and thus affects the adsorption effect^39^. Therefore, the modified red mud with a 1:4 ratio of MnO_2_ to red mud was chosen for the following experiments.

#### Effect of the adsorbent dosage

**Figure 7:**
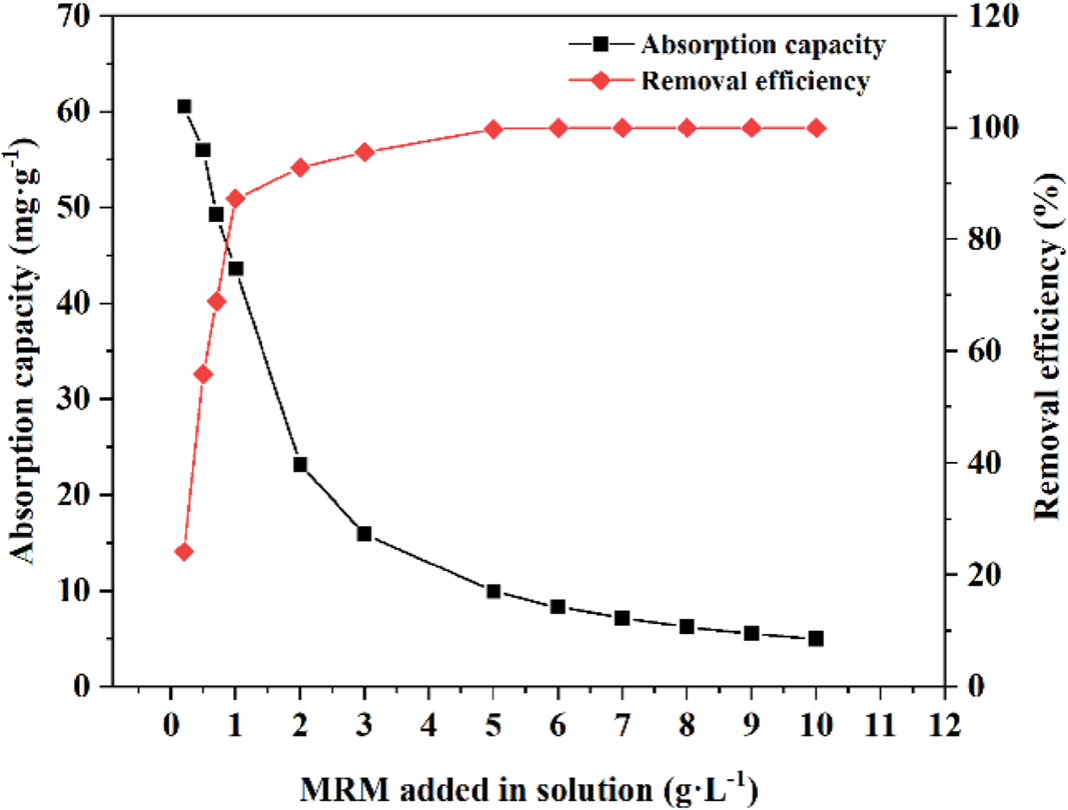
Effect of adsorbent dosage on the adsorption of Cd^2+^ on MRM at pH = 6, T = 25 °C, *t* = 240 min and initial Cd^2+^ concentration = 50 mg L^−1^.

The adsorbent dosage is one of the critical parameters to determine the adsorption capacity of the material at a fixed initial concentration. The adsorption capacity and removal efficiency curves of Cd^2+^ on MRM are shown in Fig. 7. It can be seen from the figure that the removal efficiency of Cd^2+^ showed an apparent increasing trend in the range of 0.2–4 g L^−1^ with the increase of the MRM dosage. This is because the adsorption sites available for MRM increased with the increase of the dosage, which led to the increase of the removal efficiency. When the dosage exceeded 2 g L^−1^, the removal efficiency of Cd^2+^ increased slowly and finally reached equilibrium. At the same time, the adsorption capacity of MRM decreased with the increase of adsorbent dosage. In general, when the action of adsorbent and heavy metal ions reached equilibrium, the removal rate no longer increased. Continuing to increase the dosage of adsorbent will reduce the adsorption efficiency of adsorbent per unit mass. Therefore, to save materials and consider the adsorption capacity and removal efficiency, the optimal dosage of adsorbent was determined to be 1 g L^−1^ for further experiments.

#### Effect of initial pH

**Figure 8:**
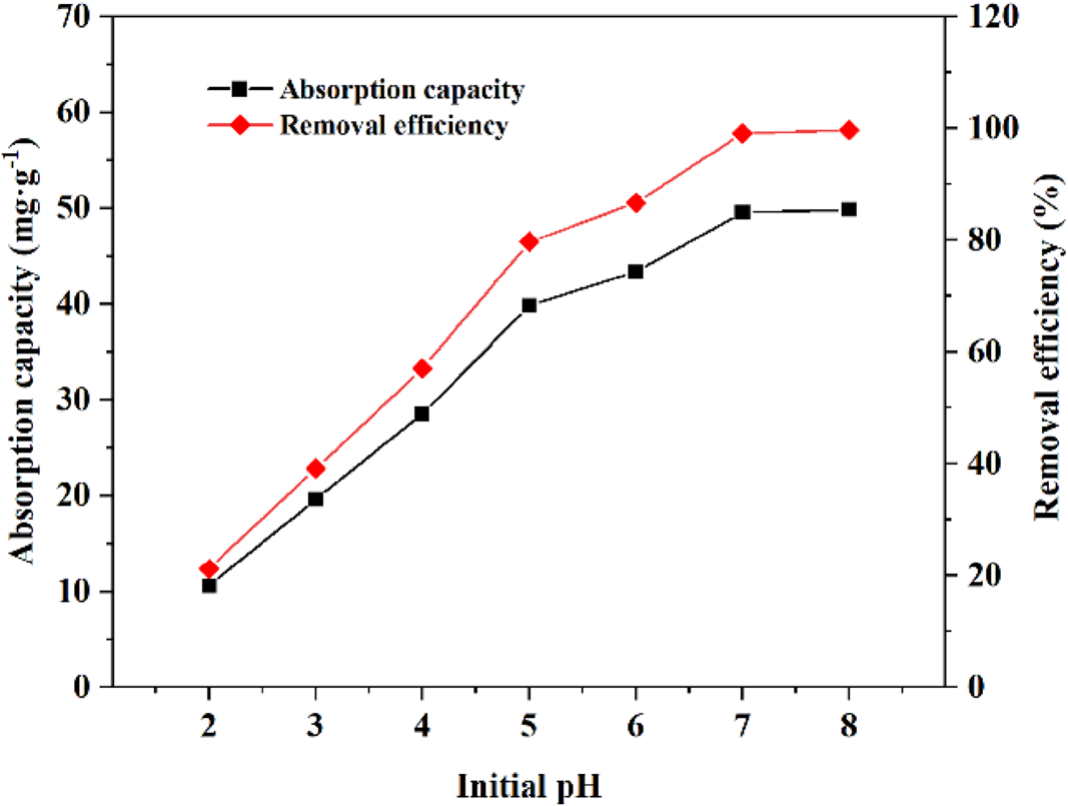
Effect of initial pH on the adsorption of Cd^2+^ on MRM at T = 25 °C, dosage = 1 g L^−1^, *t* = 240 min and initial Cd^2+^ concentration = 50 mg L^−1^.

The pH value is one of the most critical parameters to control the heavy metal adsorption process because it can influence the surface properties of the adsorbent and the ionic species in the solution. The effect of pH on the adsorption performance of MRM was analysed by varying the pH (2–8) and the results are shown in Fig. 8. The initial pH has a significant effect on the adsorption effect of Cd^2+^, and the general rule is that the adsorption rate increases with the increase of pH value. The removal rate of Cd^2+^ increased from 21.18 to 86.70% when the pH was varied in the range of 2 to 6, and even the removal rate was close to 100% at pH 7 and 8.

At lower pH (2–4), the presence of a higher concentration of H^+^ in the solution caused the accumulation of a large amount of H^+^ around MRM, which in turn led to a positive charge of manganese hydroxyl functional group due to protonation, as shown by the reaction equation in Eq. (1)^40^. The electrostatic repulsion was produced between free Cd^2+^ in solution and positively charged MRM^41^. At the same time, high concentrations of H^+^ competed with Cd^2+^ for adsorption, resulting in a relatively low amount of adsorbed Cd^2+^. While at higher pH (4–6), there was less H^+^ in the solution and the competition for adsorption was weaker. And the manganese hydroxyl functional group underwent deprotonation and became negatively charged (Eq. (2)), generating electrostatic repulsion with Cd^2+^. The resulting result is an increase in MRM adsorption efficiency. When pH > 7.4, Cd^2+^ starts to produce precipitation, so the effect of pH on the adsorption performance of MRM under alkaline conditions is not discussed in this study.

1 $$\mathrm{MnOH}+{\mathrm{H}}^{+}\iff {\mathrm{MnOH}}_{2}^{+}$$

2 $$\mathrm{MnOH}+{\mathrm{OH}}^{-}\iff {\mathrm{MnO}}^{-}+{\mathrm{H}}_{2}\mathrm{O}$$

**Figure 9:**
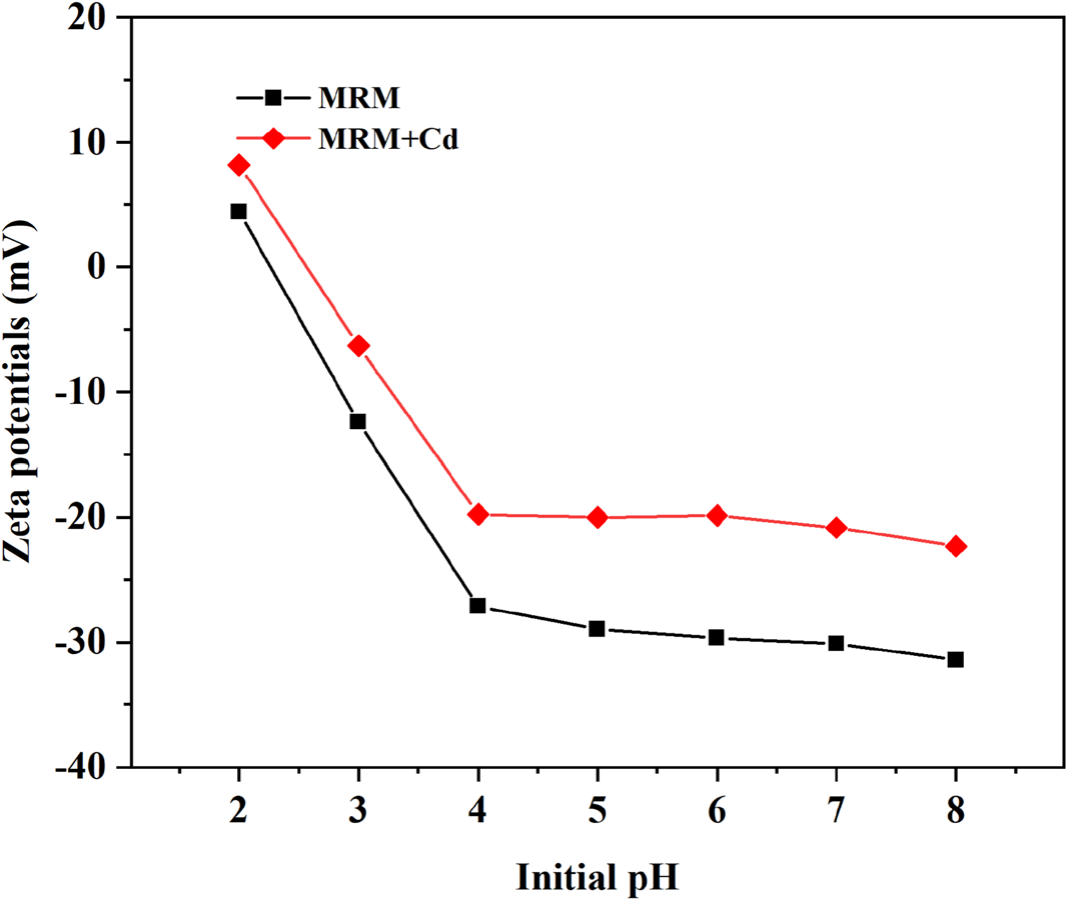
Zeta potentials of MBR and MBR-Cd (II) at various pH values.

The analysis of the Zeta potential of MRM at different pH conditions is shown in Fig. 9. It can be seen from the figure that before adsorption, the charge of MRM gradually increases with the increase of solution pH. The pH corresponding to when the zeta potential is zero is the isoelectric point of MRM (pHpzc = 2.2). After adsorption, the zeta potential of MRM decreases with the increase of pH and gradually stabilizes after pH > 4. From this, we can infer that Cd^2+^ can be adsorbed and neutralize the negative charge on the MRM surface or form metal complexes^42^.

#### Effect of coexisting cations

**Figure 10:**
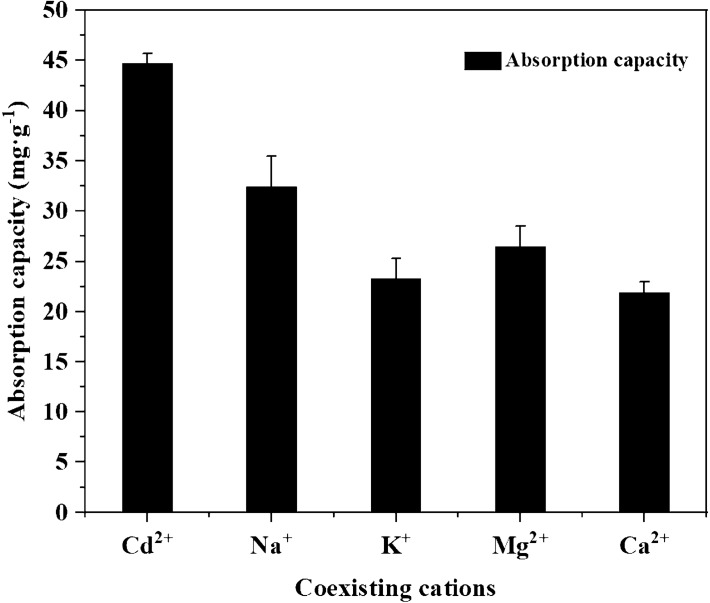
Effect of coexisting cations on the adsorption of Cd^2+^ on MRM at pH = 6, T = 25 °C, dosage = 1 g L^−1^, *t* = 240 min and initial Cd^2+^ concentration = 50 mg L^−1^.

The simulated test wastewater in this study does not contain other ions, while metal cations such as Na^+^, K^+^, Ca^2+^ and Mg^2+^ are very common in natural waters or actual wastewater and easily exhibit competitive adsorption with the target heavy metal ions^41^. The effects of different competing ions such as Na^+^, K^+^, Ca^2+^ and Mg^2+^ on Cd^2+^ removal were investigated at an initial Cd^2+^ concentration of 50 mg L^−1^ and a coexisting ion concentration of 0.2 mmol L^−1^. The effect of coexisting ions on the adsorption of Cd^2+^ by MRM is shown in Fig. 10. The results showed that Na^+^, K^+^, Ca^2+^ and Mg^2+^ in the solution all decreased the adsorption of Cd^2+^ by MRM. This is because the presence of these cations in solution competes with Cd^2+^ for the limited adsorption sites on the surface of MRM, thus inhibiting the adsorption of Cd^2+^ on MRM.

Compared with the solution without the addition of any coexisting cations, the inhibition magnitude of Cd^2+^ adsorption by the same concentration of cations was in the order of Ca^2+^ > Mg^2+^ > K^+^ > Na^+^, with Ca^2+^ having the most substantial inhibition effect. This is due to the similar structure of Ca^2+^ and Cd^2+^. The relatively small hydrated ionic radius of Ca^2+^ makes it more easily to compete with Cd^2+^ for adsorption sites, and Ca^2+^ ions can rapidly bind to the active functional groups on the MRM^43,44^. More competing ions and impurities may exist in actual industrial wastewater and natural water bodies, such as many fibres, inorganic salts and pigments in paper industry wastewater. Therefore, the practical application of MRM requires further research on its removal effect on different types of wastewater.

#### Effect of the contact time and kinetics

**Figure 11:**
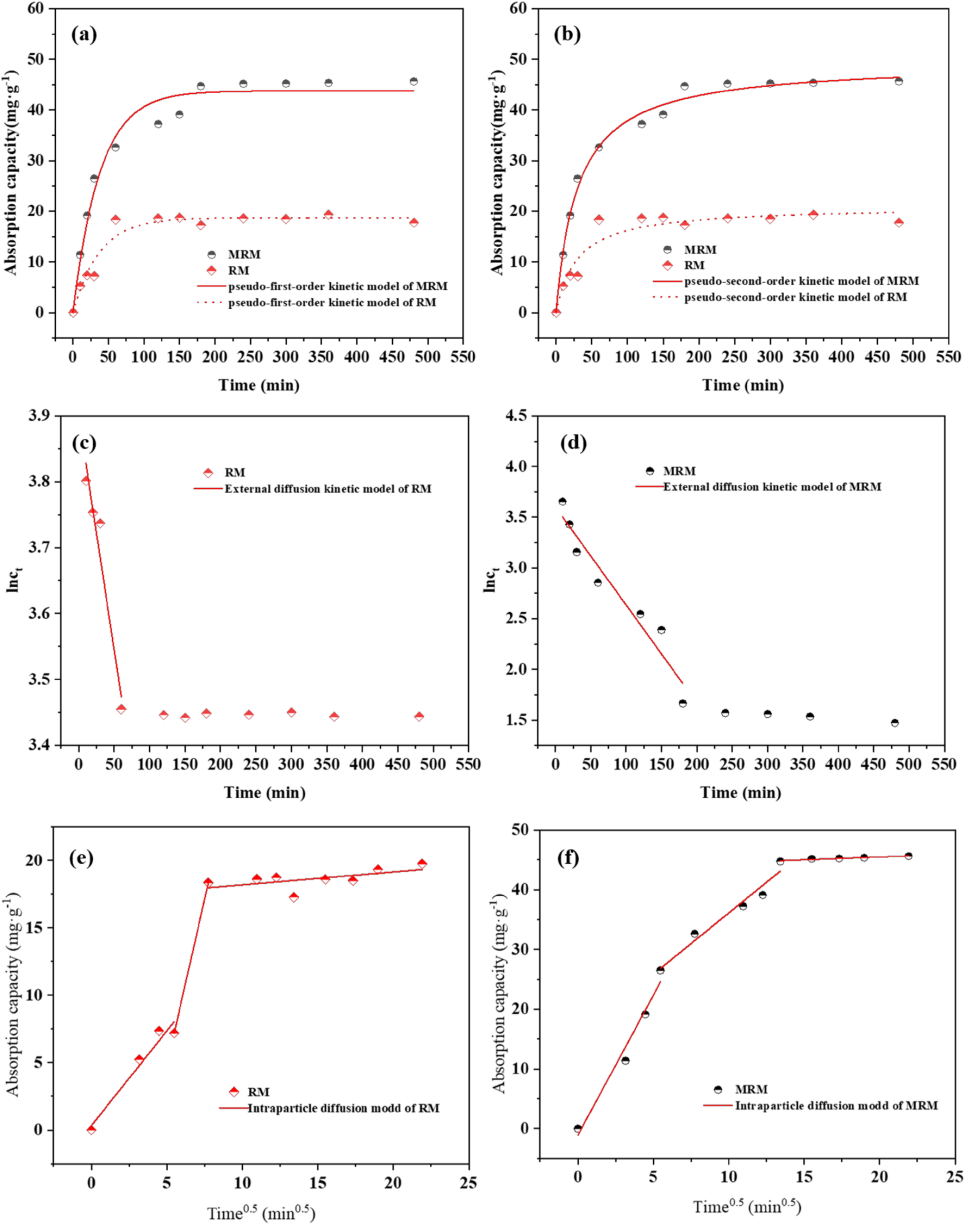
The pseudo-first-order (**a**) and pseudo-second-order (**b**) kinetic models for Cd^2+^ adsorption on MRM and RM; The external diffusion model of Cd^2+^ adsorption on MRM (**c**) and RM (**d**); The intra-particle diffusion model (**e**) of Cd^2+^ adsorption on MRM (**e**) and RM (**f**).

Samples were taken for Cd^2+^ concentration at contact times of 10, 20, 30, 60, 90, 120, 150, 180, 240, 300, 360, 420, 480 min. The effects of contact time on the adsorption performance of MRM and RM are shown in Fig. 11a. The adsorption capacity of both RM and MRM for Cd^2+^ increased with time, but the adsorption capacity of MRM was always more significant than that of RM. RM reached saturation at the contact time of 60 min, and the adsorption capacity was maintained at 18 mg g^−1^ or less. The adsorption process of Cd^2+^ on MRM can be divided into two stages: the time at 0–180 min is the surface rapid adsorption stage, and the adsorption capacity of MRM increases to 44.72 mg g^−1^; after contact time more than 180 min, the adsorption of MRM on Cd^2+^ entered the slow adsorption stage, the adsorption capacity maintained at about 45 mg g^−1^, indicating that the adsorption equilibrium has been reached. This is because, at the early stage of adsorption, there are more free adsorption sites on the surface of MRM, the concentration gradient is large, and the adsorption rate is fast. However, with the increase of contact time, the number of free adsorption sites on the surface of MRM gradually becomes less, and the Cd^2+^ already adsorbed on MRM diffuses into the interior of the material along with the pore size, leading to the increasing diffusion resistance and the decrease of adsorption efficiency, and finally reaches the adsorption equilibrium.

The adsorption kinetic model is one of the essential tools to study the adsorption mechanism. The adsorption mechanism of the adsorbent is explored by studying the rate of solute adsorption by the adsorbent and fitting the data using a kinetic model. This study used pseudo-first-order, pseudo-second-order, external diffusion kinetic, and intraparticle diffusion models to fit the obtained adsorption data. The specific equations are as follows. The correlation coefficient was used to determine the fitting effect, and the fitting results are shown in Table 2.

**Table 2:** Fitting parameters for the adsorption kinetic models.

Kinetic models and parameters	Parameters	MRM	RM
Pseudo-first order model	*k*_*1*_ (g mg^−1^ min^−1^)	0.0264	0.0270
	*q*_*e*_ (mg g^−1^)	43.7659	18.7008
	R^2^	0.9733	0.9455
Pseudo-second order model	*k*_*2*_ (g mg^−1^ min^−1^)	0.0007	0.0016
	*q*_*e*_ (mg g^−1^)	46.3611	20.9422
	R^2^	0.9904	0.9218
External diffusion model	*k*_*ext*_	0.0097	0.0071
	R^2^	0.9223	0.9209
Intraparticle diffusion model	*k*_*p,1*_ (mg g^−1^ min^1/2^)	4.4093	1.4095
	R^2^	0.9723	0.9335
	*k*_*p,2*_ (mg g^−1^ min^1/2^)	1.9492	4.9168
	R^2^	0.8618	1.0000
	*k*_*p,3*_ (mg g^−1^ min^1/2^)	0.0989	0.0956
Experimental	R^2^	0.8896	0.2605
	*q*_*e*_ (mg g^−1^)	45.65	18.75

The pseudo-first-order kinetic model:

3 $${q}_{t}={q}_{e}\left(1-\mathrm{exp}\left(-{k}_{1}\times t\right)\right)$$

The pseudo-second-order kinetic model:

4 $$\frac{t}{{q}_{t}}=\frac{1}{{k}_{2}{{q}_{e}}^{2}}+\frac{t}{{q}_{e}}$$

The external diffusion kinetic model:

5 $$\mathrm{ln}{c}_{t}=\mathrm{ln}{c}_{0}-{k}_{ext}t$$

The intraparticle diffusion model:

6 $${q}_{t}={k}_{p}\times {t}^{0.5}$$

where *q*_*t*_ (mg g^−1^) is the amount of adsorption on the adsorbent at time *t*, *q*_*e*_ (mg g^−1^) is the equilibrium Cd^2+^ adsorption capacity, *k*_*1*_ (g mg^−1^ min^−1^) and *k*_*2*_ (g mg^−1^ min^−1^) are the rate constants of pseudo-first-order adsorption and pseudo-second-order adsorption, respectively, *c*_*0*_ is the initial Cd^2+^ concentration, *c*_*t*_ is the Cd^2+^ concentration at time *t*, *k*_*ext*_ (1/min) is the constant of external diffusion, *k*_*p*_ (mg g^−1^ min^1/2^) is the intraparticle diffusion rate constant. The fitting curves obtained are shown in Fig. 11a–f.

From Table 2, the fit coefficients obtained from .the pseudo-first-order kinetic model and pseudo-second-order kinetic model were 0.9733 and 0.9904, respectively. The pseudo-second-order kinetic model was more suitable to describe the adsorption process of MRM, indicating that the adsorption process was dominated by chemisorption. This chemisorption involved electron sharing or electron transfer between the adsorbate and the adsorbent. The model fit of RM showed that the pseudo-first-order kinetic model was more consistent with the adsorption process of Cd^2+^ by RM, indicating that the adsorption process was physical adsorption. Applying the external diffusion model, we obtained the curves as Fig. 11c,d, and lnc_t_ showed a linear relationship with t in the first 180 min with R^2^ of 0.93, indicating that external diffusion was the dominant process in the initial adsorption process.

Thus, the intraparticle diffusion model analysed the rate-limiting phase on the Cd^2+^ adsorption process. According to Fig. 11e,f, q_t_ is linear for t^0.5^, but the straight line does not pass through the origin, indicating that intraparticle diffusion is not the only rate-limiting step, and there may be other steps involved in controlling the adsorption rate. The fitting curves were divided into three linear parts possessing their respective rate constants, which followed the order k_p,1_ > k_p,2_ > k_p,3_. The first linearity represents the film diffusion process with the most considerable adsorption rate. This is due to the initial adsorption process in which the vacant adsorption sites on the MRM surface can rapidly bind to Cd^2+^. The second linearity is due to the intraparticle diffusion process. In this process, the surface adsorption sites gradually decrease, and the adsorption capacity starts to approach the maximum. Moreover, the reduction of adsorption sites causes the adsorbed Cd^2+^ to enter the interior of the mesopores, generating a considerable mass transfer resistance and finally reaching adsorption equilibrium with the adsorption rate maintained at k_p,3_^45,46^.

Combining Fig. 11 and Table 2, it can be seen that MRM has higher metal adsorption capacity than RM. MRM has more active sites, larger specific surface area and pore volume, which contributes to the sufficient contact between metal ions and MRM and effectively reduces the diffusion resistance of metal ions in solution. All these results indicate that the modification of biochar by MnO_2_ is necessary and practical.

#### Effect of the initial Cd^2+^ concentration and adsorption isotherm

**Figure 12:**
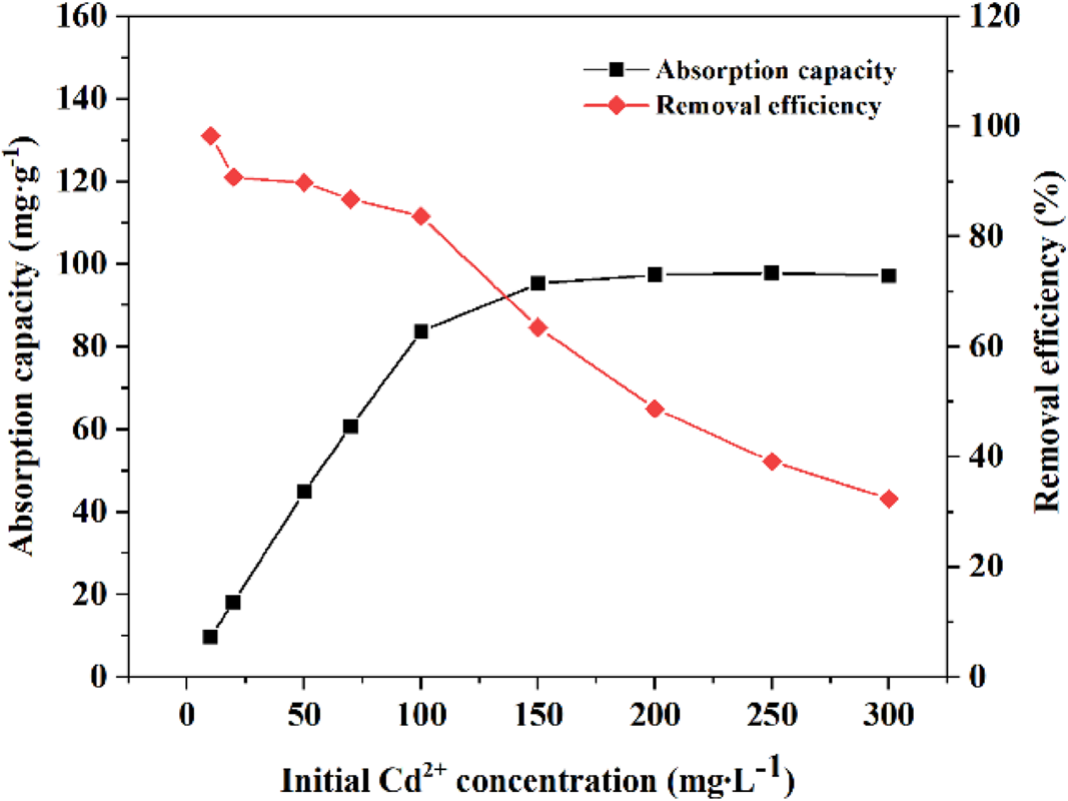
Effect of the initial Cd^2+^ concentration on the adsorption of Cd^2+^ on MRM at pH = 6, T = 25 °C, *t* = 240 min and dosage = 1 g L^−1^.

The effect of the initial concentration of Cd^2+^ on the adsorption effect of MRM is shown in Fig. 12. As can be seen from the figure, when the initial concentration of Cd^2+^ was low (10–100 mg L^−1^), the adsorption capacity of Cd^2+^ increased rapidly with the increase of concentration, and the removal rate decreased continuously. This is because there are enough adsorption sites on the MRM surface for Cd^2+^ adsorption when the initial concentration of heavy metals is low. At the initial concentration of Cd^2+^ higher than 150 mg L^−1^, the adsorption capacity of Cd^2+^ was unchanged due to the limited effective adsorption sites, and the maximum adsorption capacity of Cd^2+^ is 97.85 mg g^−1^.

**Figure 13:**
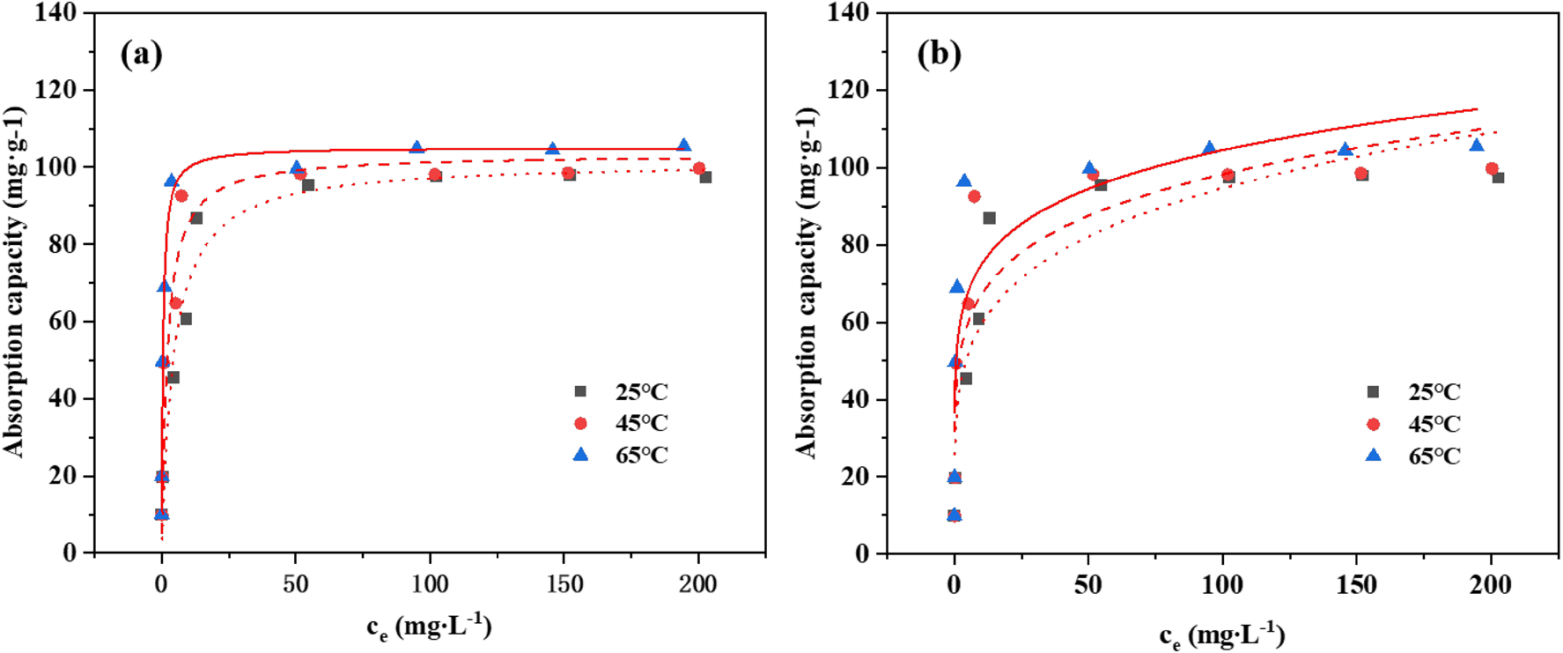
Isotherm models for Cd^2+^ adsorption onto MRM: (**a**) Langmuir isotherm model and (**b**) Freundlich isotherm model at T = 25–65 °C, Cd^2+^ concentration = 0–300 mg L^−1^, dosage = 0.1 g L^−1^, pH = 6, and contact time = 240 min.

The adsorption patterns of MRM on Cd^2+^ were investigated at three temperatures of 25 °C, 45 °C and 65 °C, as shown in Fig. 13. The common isothermal adsorption models are mainly Langmuir model and Freundich model, which were fitted to the data, and the fitted parameters are shown in Table 3.

**Table 3:** Fitting parameters for the adsorption isotherm models of MRM.

Isotherm models	Parameters	Temperature		
		25 °C	45 °C	65 °C
Langmuir model	*K*_*L*_ (mg^−1^)	0.1623	0.3054	2.4401
	*q*_*m*_ (mgg^−1^)	103.5857	116.0454	118.4734
	R^2^	0.9796	0.9242	0.9200
	*R*_*L*_	0.1097	0.0614	0.0081
Freundlich model	*K*_*F*_ (mg^1−1/n^ L^1/n^ g^−1^)	37.0624	45.6501	53.5279
	1/*n*	0.2036	0.1665	0.1452
	R^2^	0.8537	0.7823	0.7529

The Langmuir model:

7 $${q}_{e}=\frac{{q}_{m}{K}_{L}{c}_{e}}{\left(1+{K}_{L}{c}_{e}\right)}$$

where *q*_*e*_ (mg g^−1^) is the equilibrium Cd^2+^ adsorption capacity, *q*_*m*_ (mg g^−1^) is the maximum Cd^2+^ adsorption capacity, *c*_*e*_ is the equilibrium Cd^2+^ concentration of the solution, and *K*_*L*_ (L mg^−1^) is the Langmuir adsorption constant. The essential characteristics of the Langmuir isotherm can be expressed in terms of the dimensionless constant separation factor or equilibrium parameter R_L_, which is expressed as follows^47^.

8 $${R}_{L}=\frac{1}{\left(1+{K}_{L}{c}_{0}\right)}$$

where *c*_*0*_ is the initial Cd^2+^ concentration and *K*_*L*_ (L mg^−1^) is the Langmuir adsorption constant. For unfavorable adsorption, R_L_ > 1; when R_L_ = 1, linear adsorption happens; R_L_ = 0 describe irreversible adsorption; favorable adsorption occurs while 0 < R_L_ < 1^14^.

The Freundlich model:

9 $${q}_{e}={K}_{F}{{c}_{e}}^{\left(1/n\right)}$$

where *q*_*e*_ (mg g^−1^) is the equilibrium Cd^2+^ adsorption capacity, *c*_*e*_ is the equilibrium Cd^2+^ concentration of the solution, *n* is the Freundlich exponent, and *K*_*F*_ (mg ^1−1/n^ L^1/n^ g^−1^) is Freundlich adsorption constant. 1/n value indicates the strength of the effect of concentration on adsorption, with 1 < n < 10 indicating favourable adsorption^35,48^.

From the fitted parameters in the Table 3, it can be seen that the Langmuir model better reflected the isotherms of the three tesmperatures than the Freundlich model, It indicated that the adsorption of MRM on Cd^2+^ was a monomolecular layer adsorption process, and the adsorption sites on the surface of MRM were uniformly distributed. The theoretical maximum adsorption amounts obtained by the fitting were 103.5857, 116.0454 and 118.4734 mg g^−1^, similar to the experimental results. The calculations showed that the separation coefficients R_L_ in the Langmuir model were all in the range of 0 to 1, indicating that MRM was favourable for Cd^2+^ adsorption.

### Adsorption thermodynamics

To investigate the thermodynamic behavior of Cd^2+^ adsorption on MRM, the adsorption process of MRM on Cd^2+^ was studied at three temperatures of 25 °C, 45 °C and 65 °C. The thermodynamic calculations of adsorption were performed using the temperature-dependent adsorption equation and Van't Hoff equation^40^.

The Gibbs free energy (ΔG) of the adsorption reaction was calculated with the following expression.

10 $$\Delta G=-RT\mathrm{ln}{K}_{f}$$

11 $$\begin{array}{c}{K}_{f}=\frac{{q}_{e}}{{c}_{e}}\end{array}$$

where *q*_*e*_ (mg g^−1^) is the equilibrium Cd^2+^ adsorption capacity, *c*_*e*_ is the equilibrium Cd^2+^ concentration of the solution, *R* (8.314 J mol^−1^ K^−1^) is the universal gas constant, T (K) is the absolute temperature and *K*_*f*_ (L g^−1^) is the distribution coefficient.

The enthalpy change of adsorption (ΔH) and the entropy change of adsorption (ΔS) are calculated as follows:

12 $$\begin{array}{c}\mathrm{ln}{K}_{f}=-\frac{\Delta H}{RT}+\frac{\Delta S}{R}\end{array}$$

**Figure 14:**
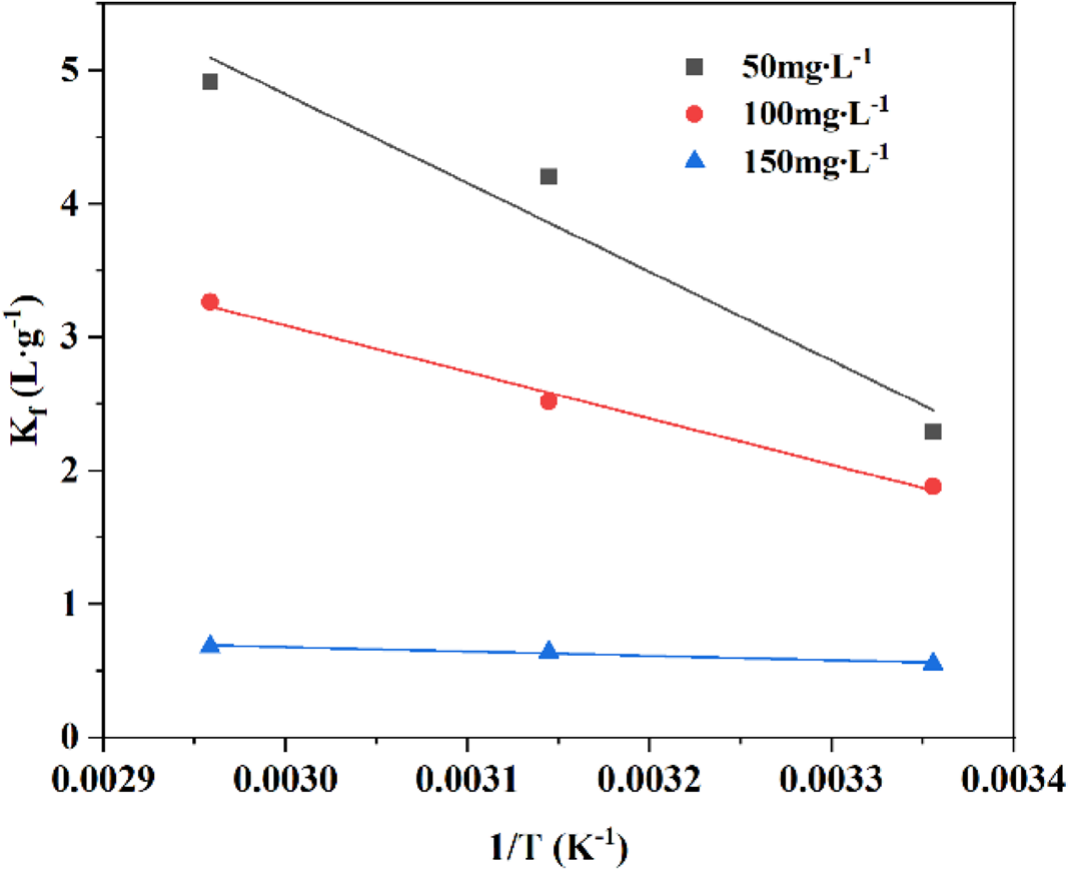
Thermodynamics model fitting of Cd^2+^ adsorption onto MRM.

The relevant thermodynamic parameters ΔG, ΔH and ΔS were calculated from the above expressions(Eqs. (10)–(12)) and Fig. 14. As shown in Table 4. ΔH values are negative, which indicates the adsorption of Cd^2+^ by MRM is a heat-taking process. The values of ΔG are always negative at different temperatures and concentrations, representing that Cd^2+^ removal is favourable and spontaneous. ΔS values are negative, indicating that the adsorption process has an increased degree of freedom between the solid–liquid interface, which may be related to the ion exchange process^49^. The positive values of ΔH and ΔS reveal that the adsorption is a heat absorption and entropy increase process, corresponding to the isotherm study.

**Table 4:** Fitting parameters for the adsorption thermodynamics model of MRM.

Initial concentration (mg L^−1^)	Δ*H* (kJ mol^−1^)	Δ*S* (J mol^−1^K^−1^)	Δ*G* (kJ mol^−1^)		
			25 °C	45 °C	65 °C
50	55.32	206.05	− 5.68	− 11.12	− 13.80
100	28.91	112.39	− 4.66	− 6.66	− 9.17
150	2.68	13.64	− 1.37	− 1.69	− 1.92

### Regeneration of MRM

**Figure 15:**
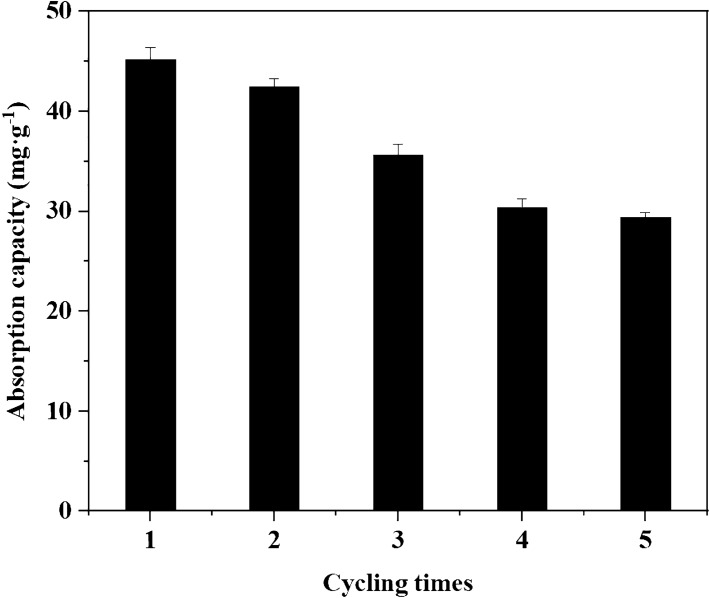
Adsorption capacity at different adsorption–desorption cycles.

The ideal and economical adsorbent should be recyclable and reusable. The variation pattern of the adsorption capacity of Cd^2+^ by MRM with the number of Cd^2+^ adsorption/desorption cycles was given in Fig. 15. The results showed that after five Cd^2+^ adsorption/desorption cycles, the adsorption amounts of Cd^2+^ by MRM samples were reduced by about 35%. Apparently, MRM had good Cd^2+^ adsorption stability and regeneration ability.

### Adsorption mechanism

**Figure 16:**
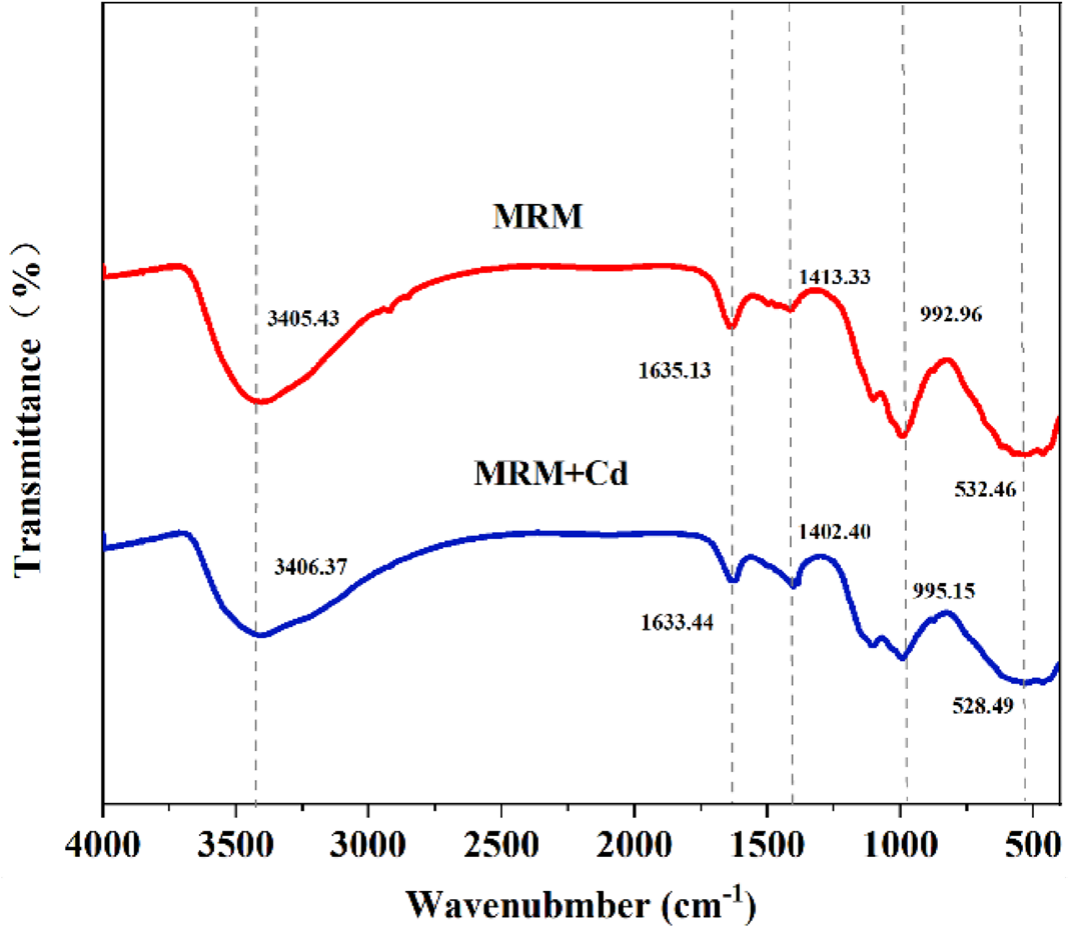
FTIR spectra of MRM before and after the adsorption of Cd^2+^.

To further determine the adsorption mechanism of Cd^2+^ on MRM, FTIR and XPS were used to analyse the elemental composition and chemical morphology of MRM before and after the adsorption of Cd^2+^. As shown in Fig. 16, no new vibrational peaks appeared in the FTIR after Cd^2+^ adsorption, but each functional group's peak intensity and wavenumber changed. The vibrational peak of –OH was weakened and shifted from 3405.43 to 3406.37 cm^−1^, which indicated that the hydroxyl group in MRM was involved in the adsorption process. In addition, the adsorption of Cd^2+^ led to a significant weakening and displacement of the Mn–OH absorption peak intensity, which was due to the entry of Cd^2+^ into the MRM and the ion exchange with the protons on the O atom of the Mn–OH group^20^. Thus, the Mn-OH group played an essential role in Cd^2+^ adsorption mainly through ion exchange^50^, demonstrating that MRM has some ion exchange capacity. The Mn–O functional group is similarly displaced, which may be due to the formation of an inner complex between the metal ion and MRM, in which a Cd–O bond was formed.

**Figure 17:**
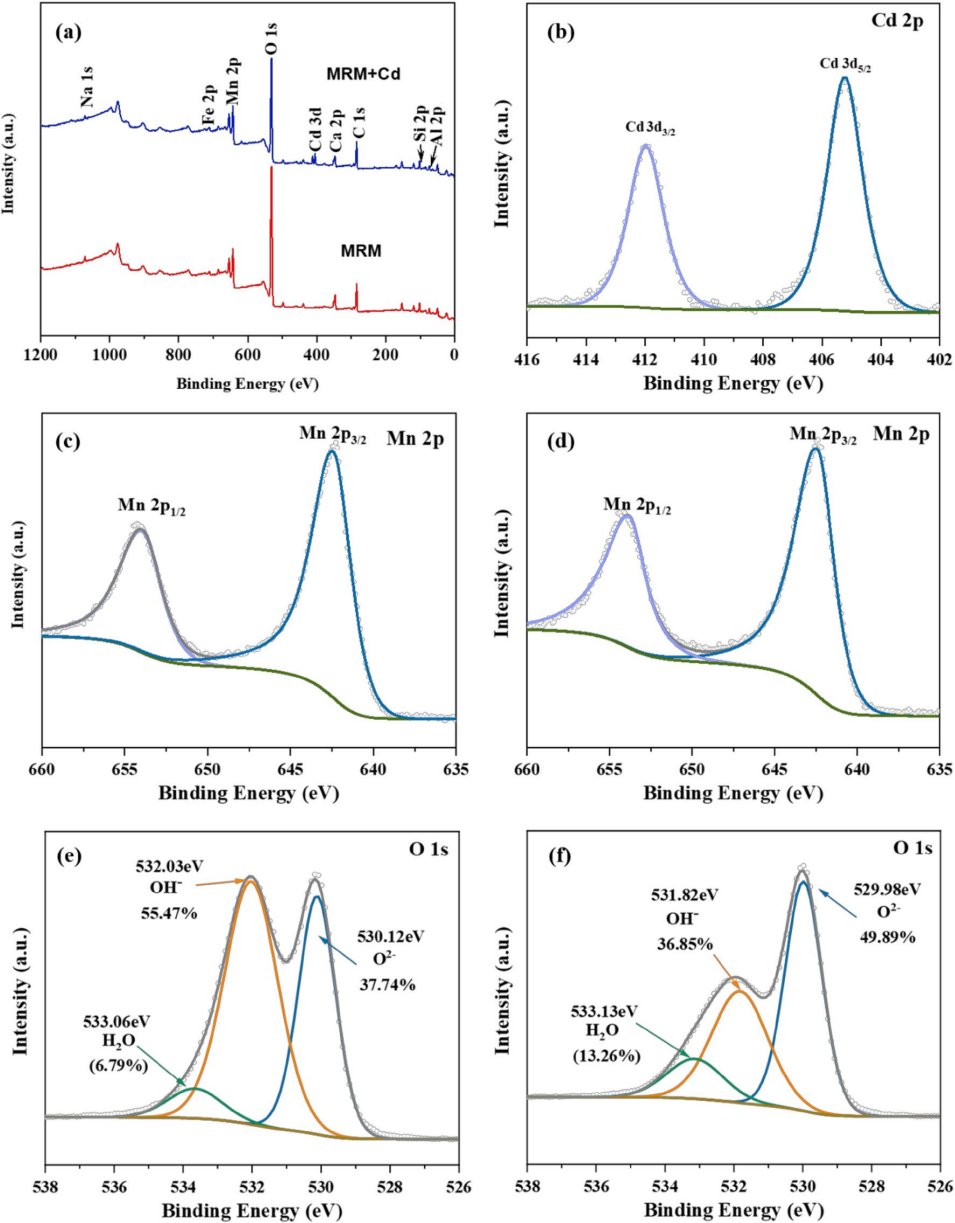
XPS results of MRM before and after adsorption (full spectra (**a**) and after the adsorption of Cd 3d (**b**), Mn 2p spectra (**c**) before and after adsorption, O 1s spectra before adsorption (**d**) and after the adsorption of Cd^2+^ (**e**).

As shown in Fig. 17a, after the adsorption of Cd^2+^, the absorption peak of Cd 3d appeared in the XPS spectrum, indicating that MRM successfully adsorbed Cd^2+^. By fitting, two peaks of Cd 3d_5/2_ and Cd 3d_3/2_ with characteristic binding energies of 405.23 eV and 411.97 eV, respectively, appeared in the XPS spectrum of Cd 3d (Fig. 17b). The appearance of the characteristic peak of Cd 3d_5/2_ indicated the formation of endogenous compounds of Cd (–OCdOH), CdCO_3_, or Cd(OH)_2_^51,52^. As shown in Fig. 17c,d, it was found that the Mn 2p spectra before and after the adsorption of MRM were very similar, with the characteristic peak binding energies of 653.88 and 642.11 eV for Mn 2p_3/2_ and Mn 2p_5/2_, respectively, indicating that Mn (IV) was always present before and after the adsorption.

According to previous reports, the O 1s profile consists of three peaks corresponding to hydroxide oxygen (OH^−^), lattice oxygen (O^2−^), and oxygen and hydroxide in molecular water (i.e., physisorbed, chemisorbed, and structural H_2_O and water in poor electrical contact with the mineral surface)^26,53^. As can be seen from Fig. 17e, before adsorption of Cd^2+^, the O 1s spectrum had three peaks at binding energies 530.112, 532.03, and 533.06 eV, corresponding to metal oxides (M–O), hydroxyl-containing metals (M–OH), and oxygen in adsorbed H_2_O, respectively. The intensity of the hydroxyl peak was significantly higher than the remaining two oxygen forms, confirming the presence of many hydroxyl groups on the surface of the MRM sample. After adsorption of Cd^2+^ (Fig. 17f), the M–OH percentage content decreased from 55.47 to 36.85%, indicating that Cd^2+^ interacts with M–OH to form hydroxy complexes (hydroxy composites) or ion exchange. In contrast, the M–O percentage content increased from 37.74 to 49.89%, indicating the formation of Mn–O–R (R: functional group) or Cd–O may be formed on the surface of MRM after adsorption. In addition, because the reaction of MRM with Cd^2+^ consumed M–OH and increased Mn–O, this process formed H_2_O, thus increasing the percentage of H_2_O^54^. In summary, it can be concluded that the mechanism of cadmium adsorption by MRM may include ion exchange, internal sphere complexation and electrostatic attraction.

### Comparison of MRM with other adsorbents for Cd^2+^ removal

To further evaluate the adsorption capacity of MRM, the Cd^2+^ removal effect of MRM and other commonly used heavy metal adsorbent materials were compared under the same experimental conditions(pH = 6, T = 25 °C, dosage = 1 g L^−1^, *t* = 240 min and initial Cd^2+^ concentration = 50 mg L^−1^.), and the obtained results are shown in Table 5. From Table 5, it can be seen that the removal of Cd^2+^ by MRM is better than many adsorbent materials reported in the literature, so MRM can be used for medium heavy metal removal in aqueous solutions, and since RM is an industrial waste and MnO_2_ is a cheap metal oxide, MRM can be prepared at a small cost.

**Table 5:** Adsorption capacities of different adsorbents for Cd^2+^ removal.

Adsorbents	Adsorption capacities (mg g^−1^)
Carbons	8.22
Zeolite	14.53
Chitosan	4.67
MRM	45.25

### Leaching metals of MRM

The leaching metal concentrations obtained from the MRM leaching test were shown in Table 6, from which we can see that the leaching toxicity of the red mud itself was weaker, and the leaching toxicity of the red mud modified by MnO_2_ was lower than the former. The MRM leaching metal concentrations were all lower than the limits of the standards for drinkingwater quality of China (GB5749-2006). Even the content of such major elements as Al and Ca was less than the standard mass value in GB 5749-2006. Therefore, the data of leached metals indicated the safety of MRM for use in aqueous environment.

**Table 6:** Leaching metals results.

Metals	Al	Mg	Ca	Cd	Pb	Cd	Cr	As	Zn	Cu	Hg	Mn
RM (mg L^−1^)	3.55	39.5	12.2	–	0.08	–	0.291	0.267	0.03	–	–	–
MRM (mg L^−1^)	0.04	–	–	–	0.01	–	0.04	–	0.01	–	–	–
GB 5749-2006 (mg L^−1^)	0.2	–	450	0.005	0.01	0.005	0.05	0.01	1.0	1.0	0.001	0.1

“–” means not required.

## Conclusions

In this study, the behavior and mechanism were conducted using amorphous MnO_2_ modified red mud to enhance the removal of cadmium, At the same time, the influencing factors of the adsorption capacity of Cd^2+^ in different process conditions were studied through adsorption experiments, including the adsorption kinetics, adsorption isotherm and adsorption thermodynamics of Cd^2+^.

Based on the findings of this study, the following conclusions and recommendations are drawn:

1. MnO_2_-modified red mud effectively improved the specific surface area and pore capacity and good adsorption effect on Cd^2+^. The adsorption performance of MRM on Cd^2+^ was affected by the ratio of MnO_2_ and red mud, pH, dosage, initial concentration of Cd^2+^, coexisting ions and contact time.
2. The pseudo-first-order kinetic model is more suitable than the pseudo-second-order kinetic model to describe the adsorption process of MRM, indicating that the chemisorption of Cd^2+^ on the surface of MRM occurs through electron sharing or electron transfer. The Langmuir model had a higher fitting coefficient than the Freundlich model, indicating that the adsorption of MRM on Cd^2+^ was a monomolecular layer adsorption process, and the surface adsorption sites are uniformly distributed. The fitted theoretical maximum adsorption amount is 103.5857 mg g^−1^, and the separation coefficient R_L_ is in the range of 0 to 1, indicating that MRM was favourable for Cd^2+^ adsorption. The thermodynamic adsorption study indicated that the adsorption reaction was a spontaneous heat absorption process.
3. Combined XPS and FTIR studies, the adsorption mechanism of MRM on Cd^2+^ was speculated to be that MRM relied on the ion exchange, internal sphere complexation and electrostatic attraction of its surface hydroxyl groups to achieve the adsorption of Cd^2+^.
4. Using MnO_2_ to modify red mud not only provides a good method to recover red mud as a waste water treatment material, but also greatly improves the adsorption performance of the red mud, which can be used as an adsorbent to treat cadmium-containing wastewater.

## Materials and methods

### Sources of the RM and reagents

The original red mud was obtained from an aluminium plant in Chongqing (Chongqing Municipality, China), where industrial alumina refining is the combined process. The main chemical composition of red mud included: CaO (30.32%), SiO_2_ (21.42%), Fe_2_O_3_ (14.90% ), Al_2_O_3_ (10.96%), Na_2_O (7.03%), TiO_2_ (5.60%), MgO (0.58%), K_2_O (0.26%), with a burn vector of 6.4%.The raw materials were crushed and passed through a 100 mesh sieve. The sample was dried in an oven (DHG-9240A, Shenzhen) at 105 °C for 48 h, sealed and stored in plastic bags as experimental raw materials, and named RM.

Potassium permanganate (KMnO_4_), manganese sulfate monohydrate (MnSO_4_·H_2_O), cadmium nitrate tetrahydrate (Cd(NO_3_)_2_·4H_2_O), nitric acid (HNO_3_), sodium hydroxide (NaOH), calcium chloride (CaCl_2_), magnesium chloride (MgCl_2_), potassium chloride (KCl) and sodium chloride (NaCl), are analytically pure and used without further purification. The water used for the experiments was deionised water.

### Preparation of MRM

**Figure 18:**
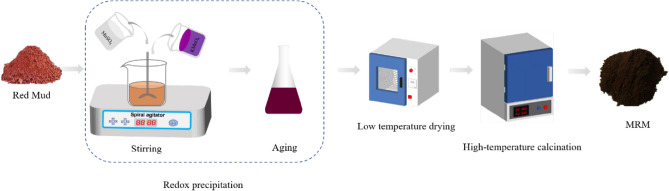
Preparation process of modified red mud.

Manganese dioxide modified red mud was prepared by the redox precipitation method using KMnO_4_ and MnSO_4_·H_2_O as manganese sources. The detailed preparation procedures are as follows.

i. We selected 0.01 mol as the initial quantity of reactants and accurately weighed 1.581 g KMnO_4_ and 1.690 g MnSO4·H_2_O ((KMnO_4_/MnSO4·H_2_O molar ratio = 1:1) and dissolved them in 100 mL of deionised water, respectively. A certain amount of red mud (MnO_2_:RM mass ratio = 1:1, 1:2, 1:3, 1:4, 1:5, 1:6) was added to a beaker containing 30 ml of deionised water to form a suspension of red mud. KMnO_4_ aqueous solution was added to the red mud suspension and stirred at 300 rpm in a constant temperature chamber for 0.5 h to make full contact between the two. Then MnSO_4_ solution was added to the above-mixed solution, and the dark brown precipitation of MnO_2_ in the suspension was produced according to Eq. (13).
  13 $$\begin{array}{c}3{\mathrm{Mn}}^{2+}+3{\mathrm{MnO}}_{4}^{-}+2{\mathrm{H}}_{2} {\text{O}}=5\text{Mn}{\mathrm{O}}_{2}\downarrow +4{\mathrm{H}}^{+}\end{array}$$
ii. After stirring at constant temperature for 0.5 h, the solution was placed in a constant temperature oscillator (35 °C, 200 rpm) for 2 h. The solution was aged for 24 h. After it cooled, it was washed and centrifuged (4000 rpm) several times to remove the residual KMnO_4_ and MnSO_4_, and finally dried at 60 °C for 8 h. The dried sample was calcined in a muffle furnace at 400 °C for 3 h to obtain the MRM sample. After cooling, it was ground into powder and stored in a container box for further experimental study. Preparation flow chart is as follows in Fig. 18.

### Characterisation analysis

In this study, the crystal phase and composition of the sample was analysed by the X-ray diffractometer (XRD, RIGAKU, Japan), with the conditions of Cu Kα as the radiation, λ = 1.5406 Å, and scanning speed of 5° min^−1^. The scanning electron microscope (SEM) and energy-dispersive spectroscopic (EDS, JSM-7500F, Japan) were obtained to investigate surface morphology and elemental composition changes of the samples. The specific surface area of the samples was measured by N_2_ adsorption–desorption experiments at 200 °C with a degassing time of 6 h using a Mike 2460 physisorption instrument and calculated by the Brunauer–Emmett–Teller (BET). The pore size of the sample was measured and calculated by Barret-Joyner-Halender (BJH) method in the N_2_ adsorption–desorption experiments. Fourier transform infrared spectrometer (FTIR, Nicolet 670, USA) was used to measure the infrared spectra of the samples and analyse the functional groups contained in the samples. In addition, the samples' X-ray photoelectron spectroscopy (XPS, Thermo Scientific K-Alpha, USA) was made with Al Kα (hv = 1486.6 eV) rays as the excitation source corrected for C1s = 284.80 eV binding energy to investigate the adsorption mechanism further.

### Batch adsorption and detection of Cd^2+^

Batch adsorption experiments investigated the adsorption performance of MRM on Cd^2+^. Cd(NO_3_)_2_·4H_2_O was used as the cadmium source, and 1.386 g of Cd(NO_3_)_2_·4H_2_O was weighed and dissolved in 500 ml of deionised water to produce 1000 mg L^−1^ of cadmium stock solution. In this study, the effects of different ratios of MnO_2_ and red mud, dosage, initial pH, initial Cd^2+^ concentration, coexisting ions, contact time and temperature on the adsorption performance of the materials were investigated sequentially. A certain amount of MRM was added to a 250 mL conical flask containing 100 mL Cd^2+^ solution and then shaken in a shaker bath (7HZ-82, Changzh·ou) for 4 h at 200 rpm. The temperature range was 25–65 °C, the dosage range was 0.02–1.0 g, the pH range was 2–8, the initial Cd^2+^ concentration range was 10–300 mg L^−1^, the contact time range was 10–480 min, and the coexisting Na^+^, K^+^, Ca^2+^ and Mg^2+^ were selected at a concentration of 0.2 mmol L^−1^. After oscillation, all samples were filtered through a 0.45 μm membrane, and the Cd^2+^ concentration in the solution was measured by atomic absorption spectrophotometer (AA 6100, Techcomp) at 228.8 nm. The removal rate and equilibrium adsorption capacity of Cd^2+^ were calculated according to Eqs. (14) and (15).

14 $$\begin{array}{c}E=\frac{{c}_{0}-{c}_{e}}{{c}_{0}}\times 100\%\end{array}$$

15 $$\begin{array}{c}{q}_{e}=\frac{\left({c}_{0}-{c}_{e}\right)V}{m}\end{array}$$

where *E* (%) is Cd^2+^ removal efficiency, *c*_*0*_ and *c*_*e*_ (mg L^−1^) are initial and equilibrium Cd^2+^ concentration in the solution, *m* (g) is the mass of MRM, *q*_*e*_ (mg g^−1^) is equilibrium Cd^2+^ adsorption capacity, and *V*(L) is solution volume.

### Leaching metal test

To examine the risk of toxic leaching of the material, the Chinese standard determination procedure (HJ/T299-2007) was adopted for MRM. A mixture of concentrated sulfuric acid and concentrated nitric acid with a mass ratio of 2:1 was added to water (about 2 drops of the mixture in 1 L of water) to prepare a leaching agent with pH 3.20 ± 0.05. The components to be measured (red mud and MRM) and leaching agent were added at a liquid–solid ratio of 10:1 (kg L^−1^), shaken for 20 h at 120 rpm and 25 °C. The number of hazardous components leached from the supernatant was determined after centrifugation.

### Desorption and regeneration studies

The MRM adsorbent was regenerated by five cycles (the adsorption test conditions were at pH = 6, T = 25 °C, dosage = 1 g L^−1^, *t* = 240 min and initial Cd^2+^ concentration = 50 mg L^−1^). The adsorbed MRM was adsorbed with 0.1 mol L^−1^ HCl to remove Cd^2+^, then washed three times with deionized water and dried to constant weight in a vacuum drying oven at 60 °C.

## Data Availability

All data included in this study are available from the corresponding author on reasonable request.
